# Delivering umbilical cord mesenchymal stem cell exosomes through hydrogel ameliorates vaginal atrophy in ovariectomized rats

**DOI:** 10.18632/aging.205302

**Published:** 2023-12-06

**Authors:** Tao Zhang, Dandan Li, Yanting Wang, Chi Zhang, Wenlan Yang, Guolan Gao

**Affiliations:** 1 Gannan Medical University, Ganzhou 341000, Jiangxi, China; 2 Savaid Medical School, University of Chinese Academy of Sciences, Huairou 101400, Beijing, China; 3 Department of Orthopedics, Peking University International Hospital, Changping 102206, Beijing, China

**Keywords:** human umbilical cord mesenchymal stem cells, exosomes, vaginal atrophy, genitourinary syndrome of menopause, hyaluronic acid hydrogel

## Abstract

Background: Menopausal and postmenopausal women often experience vaginal atrophy due to estrogen deficiency. Mesenchymal stem cell exosomes have emerged as potential therapeutic agents, capable of promoting tissue regeneration and repair. Objective: This study aimed to explore the benefits of exosomes on VK2 cells and the therapeutic effect of topical exosomal hydrogel on atrophic vaginas.

Methods: Exosomes were extracted using the high-speed centrifugation method, and their effects on VK2 cell proliferation, migration, and differentiation were observed through co-culture. The menopause model was induced by ovariectomy in rats, followed by the injection of exosome-loaded hydrogel into their vaginas. The treatment's effectiveness was evaluated by measuring vaginal epithelium thickness using HE staining, and assessing vaginal mucosa proliferation and lamina propria angiogenesis using Ki67 and anti-CD31 staining, respectively.

Results: Exosomes significantly promoted VK2 cell proliferation and migration, but had no significant effect on differentiation. The exosome hydrogel increased the expression of Ki67 and CD31, leading to a significant improvement in epithelial thickness.

Conclusions: UcMSC- ex can stimulate the proliferation and migration of VK2 cells, but do not appear to promote differentiation. Topical application of exosome hydrogel enhances vaginal epithelium thickness to a certain degree, offering a promising non-hormonal therapeutic strategy to alleviate vaginal atrophy in postmenopausal women.

## INTRODUCTION

Vaginal atrophy is a primary manifestation of the menopausal genitourinary syndrome. Primarily triggered by the decline in ovarian function and the consequential decrease in estrogen levels, it results in a fragile and thin vaginal epithelium in a low or non-estrogen state. This condition also leads to diminished local blood flow and an increase in vaginal pH, adversely affecting the sexual experience and quality of life for menopausal and postmenopausal women [1, 2]. Although systemic or topical estrogen therapy can currently improve symptoms of vaginal atrophy and augment mucosal thickness, its long-term use is controversial due to potential risks for breast cancer, endometrial cancer, and cardiovascular incidents in high-risk populations [3, 4]. Moreover, the use of lubricants and moisturizers to mitigate vaginal dryness and to alleviate pain and discomfort during sexual intercourse has not conclusively proven effective in improving vaginal histological changes [5]. Vaginal dryness, itching, and discomfort during intercourse are common adverse effects of vaginal epithelial atrophy; therefore, the restoration of the vaginal epithelium's histological structure is a critical component of symptom relief. This is particularly crucial considering many women, fearful of estrogen side effects and possessing contraindications for estrogen use, tend to favor non-hormonal products to alleviate their symptoms.

Umbilical cord mesenchymal stem cell-derived exosomes (ucMSC-ex) boast functional similarities with their parent cells, and offer several advantages including a smaller size, fewer side effects, and no ethical dilemmas. This positions them as a promising avenue for tissue regeneration therapy [6]. Exosomes, nanomembrane vesicles of 30-150 nm diameter encapsulated within a double membrane, are secreted by living cells [7]. They are laden with proteins, mRNAs, miRNAs, and lipids, enabling them to shuttle between cells and deliver biologically active substances to receptor cells to initiate a biological response [8, 9]. The regenerative abilities of exosomes derived from umbilical cord mesenchymal stem cells are well-documented, including their roles in enhancing functional repair of the spinal cord [10], promoting ovarian cell proliferation [11], and boosting angiogenesis [12]. As a major component of paracellular secretion, they are believed to effectively regenerate defective vaginal mucosa [13].

Yet, past studies have underlined challenges in exosome delivery. Specifically, intravenously or locally injected exosomes often circulate rapidly through the blood without targeting specific parts, resulting in low exosome utilization [14]. On a brighter note, with the ongoing advancements in biomaterial research, hydrogels have been identified as a potential delivery method for exosomes. They boast characteristics such as prolonging the residence time of drugs at the action site, controlling drug release rates, and have been frequently used in the local treatment of vaginal diseases [15]. Among these, the hyaluronic acid hydrogel stands out due to its excellent biocompatibility, degradability, and remarkable moisturizing properties, proving beneficial for relieving vaginal dryness in menopausal women [16, 17]. Hence, we propose it as an ideal material for vaginal drug delivery in postmenopausal women.

The objective of this study was to explore the impact of ucMSC-ex on the proliferation, migration, and differentiation of VK2 vaginal epithelium and to assess whether these influences translate positively to the vaginal epithelium *in vivo*.

## RESULTS

### Characterization of ucMSC-ex

**Figure 1:**
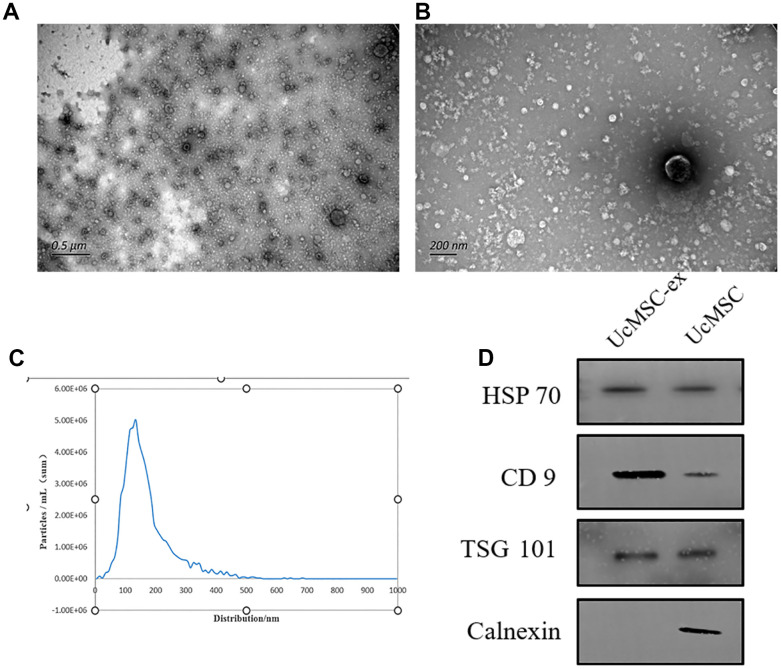
**Characterization of umbilical cord mesenchymal stem cell-derived exosomes.** (**A**, **B**) Representative transmission electron microscopy images of exosomes. (**C**) Nanoparticle tracking analysis showing the size distribution of exosomes. (**D**) Western blot analysis for the detection of specific proteins in ucMSC-ex.

UcMSC-ex were meticulously characterized using transmission electron microscopy, which revealed a distinct cup-shaped morphology and a relatively uniform diameter (Figure 1A, 1B). Through Nanoparticle Tracking Analysis (NTA), the size distribution of exosomes was demonstrated, showcasing a predominant peak at 126.7 nm that accounted for 96.6% of the main peaks (Figure 1C). Further validation with Western blotting not only confirmed the expression of hallmark exosomal markers such as CD9, HSP70, and TSG101 but also noted the absence of Calnexin (Figure 1D). These combined results underscored the successful isolation of exosomes.

### Successful uptake of exosomes by vk2 cells

**Figure 2:**
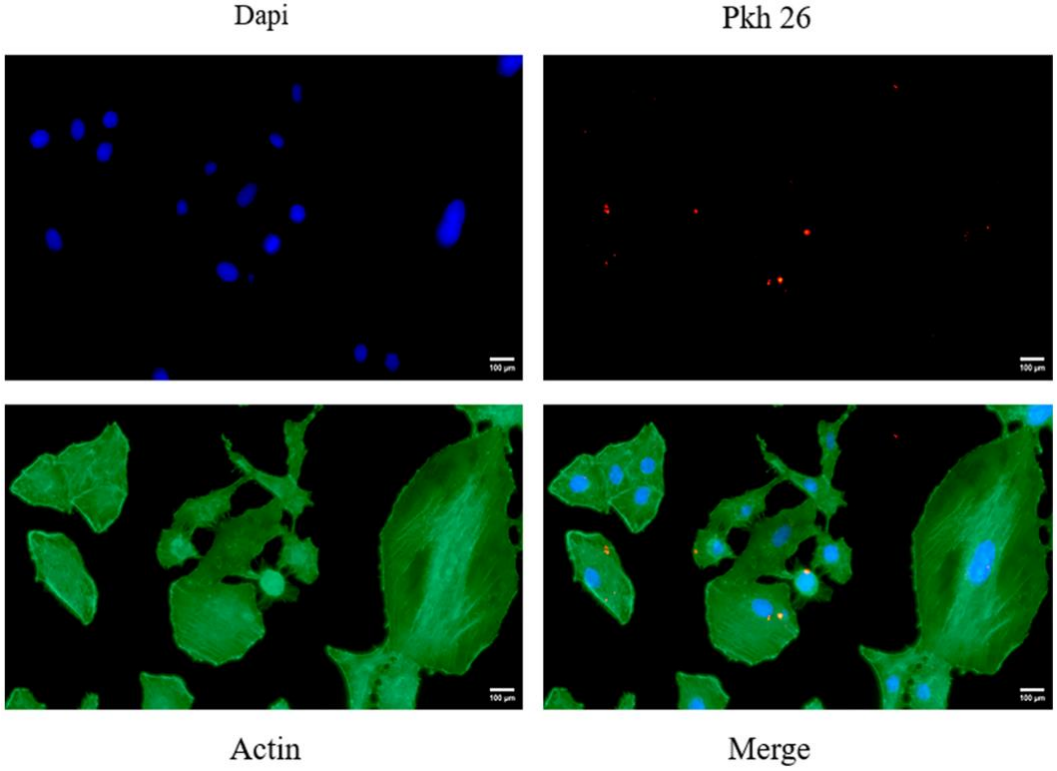
Shows the fluorescent images of VK2 cells taking up ucMSC-ex.

VK2 cells, which were stained green for the cytoskeleton and blue for the nucleus, efficiently ingested exosomes that were labeled red by PKH26, following a 12-hour co-culture. Notably, the exosomes aggregated around the nucleus, situated within the cytoskeleton (Figure 2), further confirming their successful uptake by VK2 cells.

### Exosomes promote vk2 cell migration

**Figure 3:**
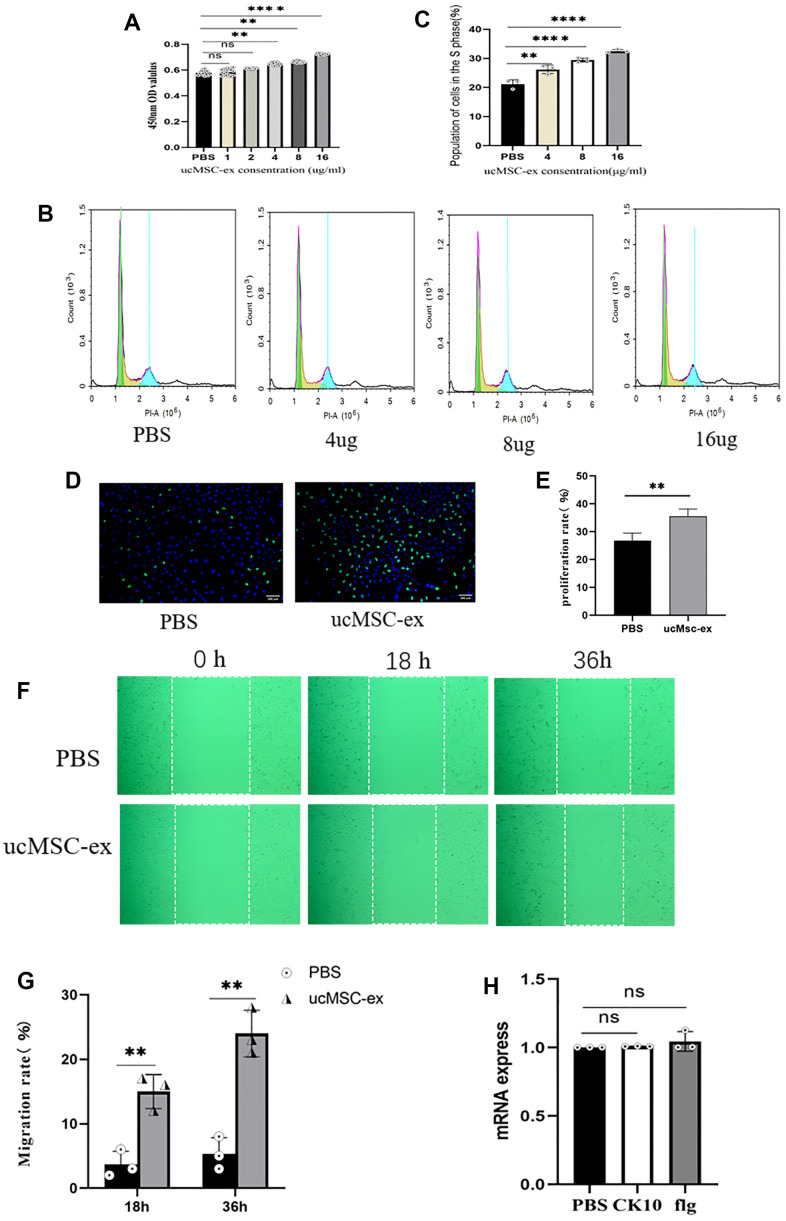
**The effects of ucMSC-Ex on VK2 cell proliferation, migration, and differentiation.** (**A**) CCK-8 assay measuring VK2 cell viability at different concentrations; (**B**) Flow cytometry analysis of cell cycle after co-culture for 18 hours; (**C**) Quantification of S-phase cell proportion; (**D**) EdU assay detecting VK2 cell proliferation after 24 hours of co-culture; (**E**) Quantitative analysis of EdU-positive cells; (**F**) Scratch assay showing ucMSC-Ex promotion of VK2 cell migration; (**G**) Quantification of cell migration; (**H**) Normalized expression levels of differentiation-related genes in ucMSC-ex treated group compared to PBS group.

Epithelial cell migration plays a pivotal role in the healing and recovery of injured tissues. As depicted in Figure 3F, when compared to the PBS control, our data reveal that exosome treatment markedly boosts the migration rate of VK2 cells. Flg and CK10 are established as essential markers for keratinocyte differentiation [18]. After normalization and subsequent qPCR analysis, a modest upregulation of these two markers was detected following exosome treatment. Nevertheless, it's essential to underscore that these observed changes did not achieve statistical significance (Figure 3H).

### Characterization of hyaluronic acid hydrogels

**Figure 4:**
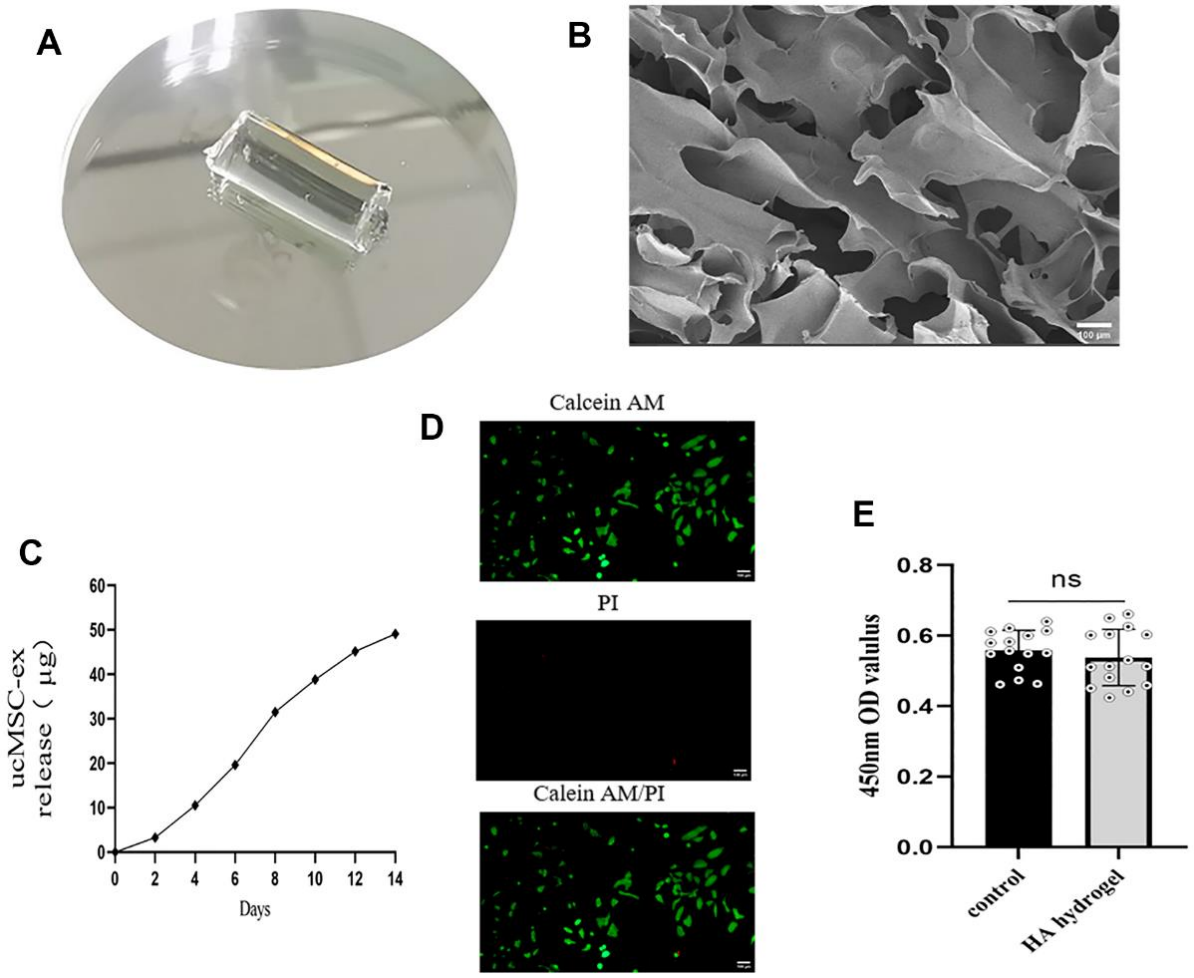
**Characterization of exosome hydrogel.** (**A**) Morphology of hyaluronic acid hydrogel post photopolymerization; (**B**) Cross-sectional view of hyaluronic acid hydrogel presenting a loose, porous structure under scanning electron microscopy; (**C**) Release curve of exosomes from the exosome hydrogel over 14 days; (**D**) Live/dead cell staining of VK2 cells cultured on the hyaluronic acid hydrogel. (**E**) Determination of cell viability by co-incubation of vk2 cells and hydrogels.

Post-photocrosslinking, the hyaluronic acid hydrogel took a cylindrical shape, measuring a diameter of 5 mm and a height of 1.0 cm (Figure 4A). SEM images revealed that the gel's interior hosted a loosely interconnected pore structure, affording ample space for accommodating exosomes (Figure 4B). As illustrated in the figure, the natural degradation of the hydrogel facilitated a slow, continuous release of the loaded exosomes (Figure 4C). To explore the biocompatibility of hyaluronic acid hydrogel, we conducted live/dead staining experiments. In these experiments, green fluorescence represented live cells stained with calcein AM, and red fluorescence indicated dead cells stained with PI (Figure 4D). The results indicated that the majority of VK2 cells remained viable after exposure to the hydrogel. Furthermore, additional CCK-8 analysis (Figure 4E) revealed no significant difference in cell viability between the two groups, demonstrating that the hyaluronic acid hydrogel exhibits excellent biocompatibility and is devoid of any cytotoxicity.

### Hematoxylin-Eosin (HE) analysis

**Figure 5:**
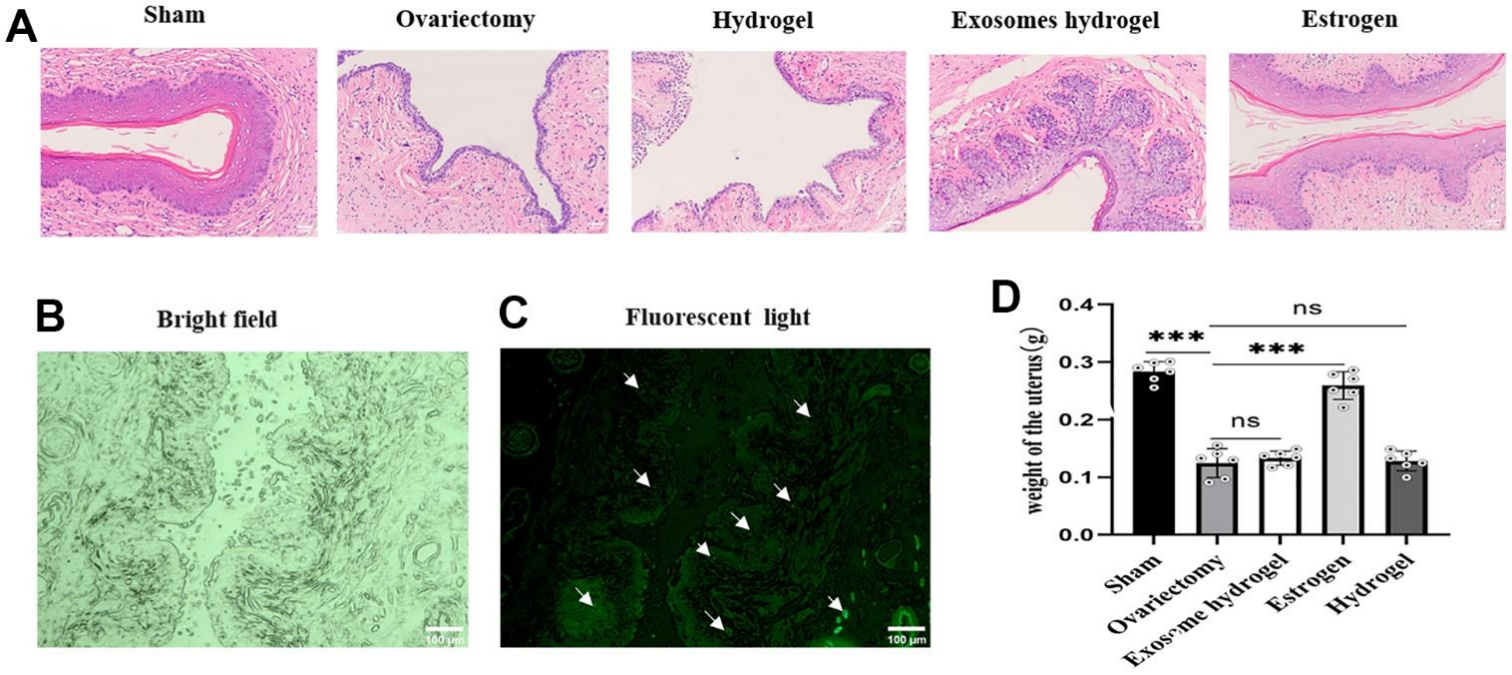
**Vaginal sections and HE staining.** (**A**) Vaginal epithelial morphology among different groups after HE staining; (**B**, **C**) Distribution of DIR-labeled exosomes in vaginal tissue; (**D**) Uterine weights in each group.

Figure 5A shows that the vaginal epithelium is the thickest in the sham operation group. The nuclei of the basal layer cells are large and deeply stained, aligned neatly in a palisade arrangement. The stratum corneum is complete, with spindle-shaped granular cells and non-nucleated squamous cells observable between the basal layer and the stratum corneum. In the ovariectomy group and hydrogel group, the epithelium is thin, the arrangement of basal cells is disordered, the number of cell layers is small, and the stratum corneum disappears. Papillary-like proliferation is observed at the vaginal base in the exosome hydrogel group, the epithelial cell layers increase in number, the thickness is considerably greater than the ovariectomy group, albeit slightly less than the sham operation group. The thickness of the vaginal mucosal cells in the estrogen treatment group and the sham operation group is similar, which is consistent with previous reports: estrogen can completely reverse the atrophy of the vaginal epithelium [19]. Moreover, when analyzing uterine weight across groups, there was no statistically significant difference observed between the exosome and hydrogel groups compared to the de-ovulated group (Figure 5D). This implies that the application of exosome hydrogel does not influence uterine weight.

### Immunohistochemistry

**Figure 6:**
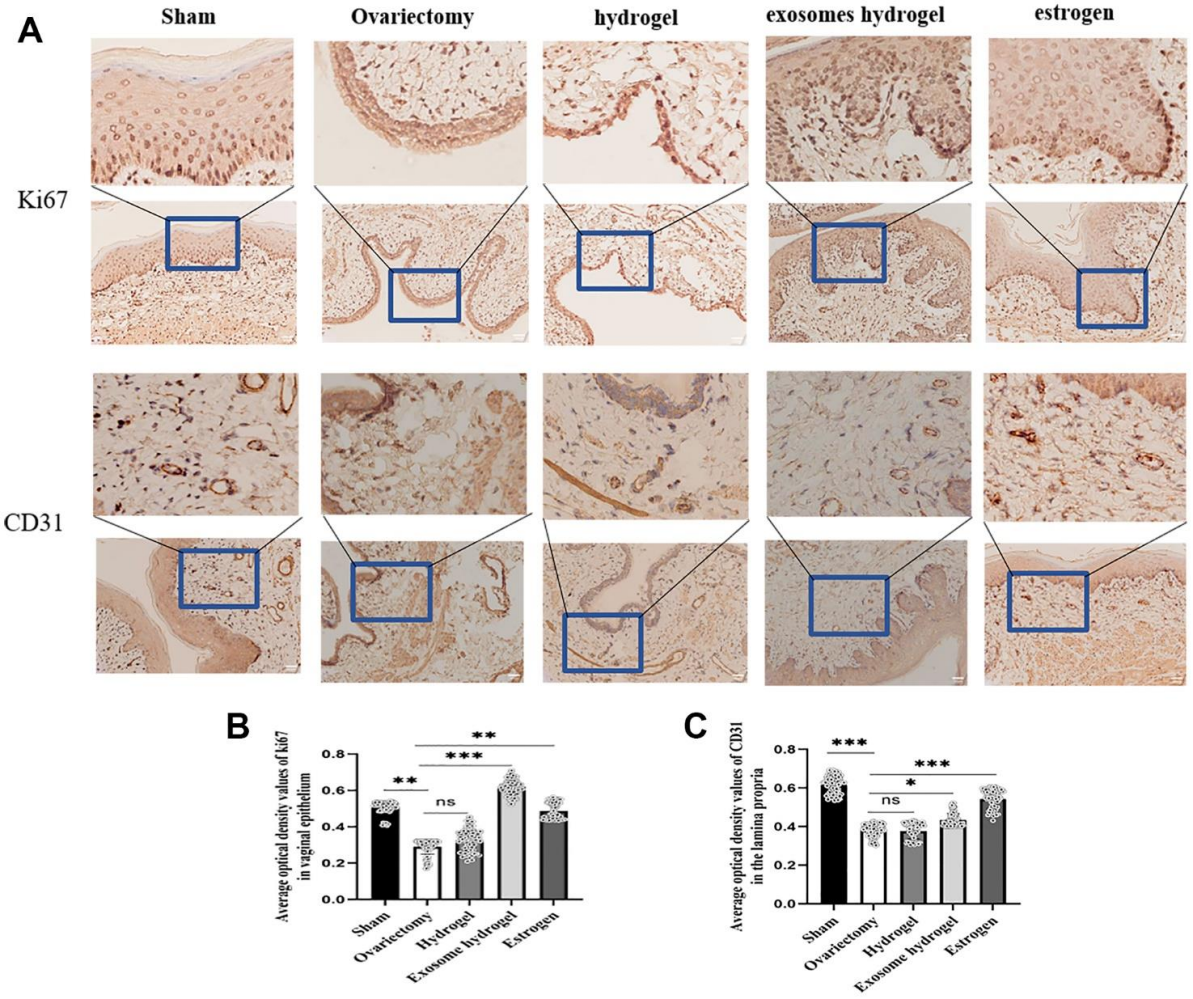
**IHC analysis.** (**A**) Average optical density analysis of ki67 and CD31 expression in the vaginal epithelium and lamina propria; (**B**, **C**) Quantification of ki67 and CD31 staining.

In an effort to ascertain the distribution of exosomes within the rat vagina, we examined vaginal slices marked with fluorescent DIR under a microscope. As can be discerned from the figure, green fluorescent-tagged exosomes were seen in the vaginal basal layer, sub-basal layer, and surrounding blood vessels, implying that exosome particles released from the hydrogel can be absorbed by tissue cells (Figure 5B, 5C). Ki67 is a classic marker of cell proliferation. Figure 6 reveals that the expression of epithelial cells in the vaginal mucosa is at its nadir in the ovariectomy group and the hydrogel group, yet, the exosome hydrogel treatment group exhibits the highest expression among all groups, with the sham operation group and the estrogen group showcasing similar expression levels. The expression level in the hydrogel treatment group mirrors that in the ovariectomy group, while it experiences a significant surge in the exosome hydrogel group, which underscores that the active proliferation of vaginal basal cells is primarily attributable to the effect of exosomes. Furthermore, we meticulously quantified the CD31-positive capillaries in the inherent layer, and as was expected, the count was the highest in the sham surgery group, trailed by the estrogen group. The quantity of CD31-positive microvessels diminished post-ovariectomy, but saw a revival following treatment with exosome hydrogel.

## DISCUSSION

In the current study, we provide evidence that exosomes derived from umbilical cord mesenchymal stem cells (MSCs) promote the proliferation and migration of VK2 cells *in vitro*, but do not significantly affect their differentiation. The role of ucMSC-ex in regenerative therapy is a topic of intense research interest [20]. A proteomic analysis of umbilical cord MSC exosomes revealed an enrichment of specific proteins such as FN1, CDC42, and MAPK1 [21]. FN1, a classical extracellular matrix component, plays a key role in regulating cell adhesion and migration [22]. CDC42, on the other hand, is instrumental in regulating cell growth and proliferation [23]. MAPK1 is implicated in diverse cellular processes, including proliferation, differentiation, transcription regulation, and development. In addition to these proteins, exosomes are rich in miRNAs, which modulate the expression of multiple genes through a complex regulatory network to produce biological effects. For example, umbilical cord MSC-derived exosomes have been reported to deliver miRNA-17-5p, exerting a pro-proliferative effect to ameliorate premature ovarian failure [24]. Furthermore, miR-100, miR-146a, and miR-221 have been implicated in the regulation of VK2 vaginal epithelial growth [25]. Although the exact mechanism through which exosomes exert their proliferative and migratory effects on VK2 cells remains unclear, it is well-established that they fulfill their function by delivering proteins and RNAs. Thus, our findings add to the growing body of evidence suggesting a promising role for umbilical cord MSC exosomes in regenerative medicine.

Intravaginal administration is a classic approach for treating both local and systemic diseases [26]. The vaginal epithelium has several key advantages as a site for drug delivery, including a large surface area, a rich blood supply, high permeability to small molecules, and avoidance of the hepatic first-pass effect [27]. Topical administration in the vagina reduces adverse effects, decreases the frequency of dosing, and offers direct therapeutic impact, alongside limiting systemic drug toxicity by releasing the drug directly to the target site [28, 29]. Enhanced therapeutic efficacy has been reported when hydrogels are loaded with exosomes or other nanoparticles, as these increase the efficiency of cellular uptake [30, 31]. Hyaluronic acid has been widely used in gynecology, notably as a moisturizer for vaginal use in women. Previous studies have also reported the promotion of endometrial regeneration through the *in-situ* delivery of apoptotic bodies derived from mesenchymal stem cells via hyaluronic acid hydrogels [32]. In our study, we designed a hyaluronic acid/exosome system that matched the shape and length of the rat vagina. The exosomes were released as the gel naturally degraded, and were then absorbed by the cells. We demonstrated that exosomes labeled with DIR were found in the basal lamina and around blood vessels, suggesting that the hyaluronic acid hydrogel successfully delivered the exosomes. Previous studies reported that hyaluronic acid alone promotes vaginal epithelial thickening [33]. However, our study did not reach a consistent conclusion on this matter, which may be due to variations in the timing, dosage, and formulation of the hyaluronic acid used. Therefore, further research is needed to understand the precise role and potential of hyaluronic acid in the context of vaginal health and disease treatment.

The vascular supply of the vaginal mucosa is associated with the distribution of estrogen receptors. Estrogen receptor alpha (ERα) is primarily found in the vaginal epithelium, stroma, and smooth muscle cells, while estrogen receptor beta (ERβ) is mainly located in vascular smooth muscle cells. Previous studies have highlighted differences in estrogen receptor expression between women of childbearing age and postmenopausal women, with a decrease or even disappearance of ERβ observed in the latter group [34, 35]. This implies that natural aging or medical interventions leading to estrogen decline can result in the degradation of vaginal blood vessels. Several studies have shown that exosomes derived from umbilical cord mesenchymal stem cells (MSCs) can promote angiogenesis by delivering numerous active substances [36–38]. In our study, we used CD31, a biomarker of endothelial cells, to quantify the number of blood vessels. We found a significant reduction in blood vessels in the lamina propria in the ovariectomy group. However, when the exosome hydrogel was administered, the number of neovascularizations increased, indicating that the exosome hydrogel may protect the atrophic vaginal mucosa through its pro-angiogenic and pro-proliferative effects.

The vaginal epithelium, a stratified and keratinized squamous epithelium, is formed and maintained by a delicate balance of epithelial basal cell proliferation and squamous differentiation. A fully stratified and keratinized vaginal epithelium mirrors the epidermal stratification of the skin. However, the proliferation and differentiation of vaginal epithelium are primarily regulated by estrogen. When estrogen binds to its receptors, it mediates the proliferation, migration to the suprabasal layer, and terminal differentiation of vaginal basal lamina cells, resulting in stratification. The activation of the Wnt signaling pathway has been demonstrated to play a crucial role in this process of proliferation and differentiation [39]. In our model, contrary to the traditional understanding, there seems to be no involvement of estrogen. Intriguingly, our study detected the highest Ki67 expression in the exosomal hydrogel group, indicative of highly active vaginal epithelial cell division. This suggests that exocytosis bolstered the proliferation of vaginal basal cells, a result that aligns with our *in-vitro* study. However, no significant promotion of differentiation was observed. Despite this, our experiments unveiled significant stratification and thickening, hinting at an alternative mechanistic path to this outcome. According to mechanobiological studies, the proliferation of newborn keratinocytes in the basal layer pressures the original basal layer keratinocytes, prompting these undifferentiated keratinocytes to differentiate. This pressure alters the original connection dynamics, allowing keratinocytes to reshape and move upward, forming stratification [40, 41].

In summary, our findings suggest that exosome hydrogels may ameliorate vaginal atrophy by promoting neovascularization in the vaginal tissue and facilitating basal cell proliferation and migration. These results offer exciting potential for the development of new therapeutic approaches to treat conditions associated with vaginal atrophy, such as postmenopausal vaginal dryness. However, further research is needed to fully understand these mechanisms and their potential clinical applications.

While the use of exosomes loaded into hydrogels has been previously explored in various disease models, our study, to the best of our knowledge, is the first to investigate exosome-mediated re-epithelialization in the context of vaginal atrophy in a menopausal model. By conducting *in-vitro* studies, we have been able to validate the effects of exosomes on VK2 cell proliferation, migration, and differentiation. Taking a step further, we utilized an animal model to confirm the re-epithelialization effect of exosomes on atrophic vaginal tissue, thus suggesting a non-hormonal therapeutic strategy for improving vaginal atrophy. However, there are still limitations to our study. Firstly, this is a short-term investigation, and we did not observe the duration over which the thickening of the vaginal mucosal epithelium, following the cessation of the exosome hydrogel system, is sustained. Consequently, it is not possible to assess its frequency and duration of use. Secondly, we did not monitor changes in the vaginal microbiota, pH levels, and enzymes before and after treatment. Histological improvements serve as the foundation of vaginal health, and the stability of vaginal microbiota, including an increase in lactobacilli and a reduction in pH, is pivotal for ameliorating menopause-related symptoms and enhancing local immunity. Furthermore, even though short-term usage of exosomes within the body appears safe, it is imperative to explore the safety of long-term utilization in the future.

## CONCLUSIONS

Our *in vitro* study revealed that exosomes promote VK2 cell proliferation in a dose-dependent manner and significantly enhance VK2 cell migration. However, they do not appear to affect epithelial cell differentiation. We have discovered that by loading exosomes into gels, the epithelial thickness can be improved to some extent. These finding sheds light on a new non-hormonal approach to the treatment of vaginal atrophy.

## MATERIALS AND METHODS

### Cell source and culture

Umbilical cord mesenchymal stem cells (ucMSCs) and vaginal epithelial cells (VK2) were obtained from the Shanghai Cell Bank (ATCC, China). The ucMSCs were cultured in a medium containing 10% fetal bovine serum (Gibco, USA), 1% dual antibiotic (streptomycin + penicillin), and low glucose DMEM. UcMSCs from 2-6 generations were evenly seeded in 15 cm dishes using the complete culture medium and incubated at 37° C with 5% CO2. The culture medium was refreshed every 2-3 days. When the cells achieved 80% confluency, the culture medium was removed, washed three times with PBS, and then replaced with a 1:1 volume ratio of low glucose DMEM and exosome-specific serum-free medium (Umbibio, China). The cells were then further incubated for 48 hours and the cell supernatant was collected.

According to the cell manufacturer's instructions, VK2 cells were cultured with 10% fetal bovine serum (Absin, China), 1% double antibiotic (streptomycin + penicillin), high glucose DMEM, under similar culture conditions as the ucMSCs.

### Isolation, identification, and labeling of exosomes

Using the method prescribed by Huilei Yu [42], the collected cell supernatants were first centrifuged at 300g for 5 minutes at 4° C to discard live cells. Subsequent centrifugation at 2000g for 30 minutes was used to remove dead cells, followed by another round at 10,000g for 30 minutes to exclude macromolecules and cell debris. The supernatant was then filtered using a 0.22 μm filter membrane and centrifuged at 100,000g for 70 minutes. The resulting precipitate was resuspended in PBS and centrifuged once more at 100,000g for 70 minutes to finally obtain a light-yellow precipitate.

A part of the exosome suspension was mixed with red PKH26 and green DIR (Umbibio, China) dyes, separately, in dark conditions. The excess dye was removed following the exosome extraction protocol, and the precipitate was resuspended in PBS to acquire the stained exosomes.

The morphology of the exosomes was studied using transmission electron microscopy, and the nanoparticle concentration was measured by counting the scattered particles using a nanoparticle tracking system.

Western blotting was carried out using exosome markers CD9 (1:1000; Absin, China), TSG101 (1:1000; Umbibio, China), HSP70 (1:1000; Affinity, China), and Calnexin (1:1000; Affinity, China). In brief, proteins were separated by gel electrophoresis (PAGE 10% Bis-Tris, Meilunbio, China) and transferred to a 0.22 μm polyvinylidene difluoride membrane (PVDF, Millipore, USA). They were then immunoblotted with a specific primary antibody, blocked with 5% skimmed milk powder, and subsequently blotted with a secondary rabbit antibody (1:3000; Affinity, China) for 1 hour at 37° C. Finally, the blots were visualized with an ultra-sensitive ECL kit (Meilunbio, China).

### Uptake of exosomes

PKH26-labeled exosomes and VK2 cells were co-incubated at 37° C for 12 hours. Subsequently, the cells were fixed with 4% paraformaldehyde, nuclei stained with DAPI (Beyotime, China), and the cytoskeleton was stained with Actin-Tracker Green (Beyotime, China). After washing with 1% Triton ×100, images were captured using a Zeiss fluorescence microscope.

### Cell viability, cell cycle, and EDU assay

Cells were seeded in 96-well plates at a density of 5 x10^3 cells/well. Control wells received PBS while experimental wells were treated with exosomes at concentrations ranging from 1μg to 16μg. After 24 hours incubation, 10μl of CCK-8 (Glpbio, USA) reagent was added to each well and incubated for an additional 2 hours. Absorbance was then measured at 450nm.

VK2 cells (5x10^5) were seeded in 6-well plates and incubated for 24 hours. Following incubation, wells received either PBS or exosomes (at concentrations of 4μg, 8μg, and 16μg). After 18 hours, cells were harvested, fixed with 75% ethanol, and stored at -20° C overnight. Subsequently, the Cell Cycle and Apoptosis Analysis Kit (Beyotime, China) was used for staining and flow cytometry analysis. Raw data were analyzed using FlowJo 10.6.2 software (FlowJo, USA).

VK2 cells in the logarithmic growth phase were seeded in 96-well plates at a density of 3x10^3 cells/well. Control wells received PBS and experimental wells received 16μg of exosomes. After 24 hours incubation, cells were labeled with 10uM EdU (Beyotime, China) for 2 hours. Cells were then fixed with 4% paraformaldehyde, permeabilized with 0.3% Triton × 100, and processed with a click reaction. Nuclei were stained with Hoechst 33342. Images were captured under a microscope, and the number of EdU-positive cells was determined. The EdU staining rate was calculated as follows: (number of EdU-positive cells/(EdU-positive cells + EdU-negative cells)) x 100%.

### Cell migration

VK2 cells were seeded in 12-well plates and allowed to reach 100% confluency. A straight scratch was made across the cell monolayer using a 200μl pipette tip. After washing with PBS, cells were cultured in serum-free high glucose DMEM medium. Control wells received PBS and experimental wells received 16μg of exosomes. Cell migration was monitored and imaged at 0, 18, and 36 hours. The scratch wound healing was calculated as follows: (initial scratch area - remaining scratch area at the time of measurement) / initial scratch area x 100%.

### Quantitative real-time polymerase chain reaction (qRT-PCR) analysis to detect cell differentiation

Cells were seeded in 6-well plates and allowed to reach 70% confluency. Control wells received PBS and experimental wells received 16μg of exosomes. After 72 hours incubation, the cells were harvested and the expression of Filaggrin and Cytokeratin 10 (Flg, CK10) was determined by qPCR. Related primer sequences are shown in Table 1.

**Table 1:** Primers for quantitative PCR analysis.

**Primer name**	**Forward primer**	**Reverse primer**
GAPDH	GGAGCGAGATCCCTCCAAAAT	GGCTGTTGTCATACTTCTCATGG
CK10	GACAAAGTTCGGGCTCTGGA	CCCCTGATGTGAGTTGCCAT
Flg	ATCTGAGGGCACTGAAAGGC	CACTTCCGTGCTGAGAGTGT

Abbreviations: GAPDH, Glyceraldehyde-3-phosphate dehydrogenase; CK10, Cytokeratin 10; Flg, Filaggrin.

Total RNA was extracted from cells using the RNA Isolation Kit V2 (Vazyme, China), which was then reverse transcribed to cDNA using the 4x reverse Transcription Master Mix (Vazyme, China). qPCR was conducted using the SYBR Green qPCR Master Mix (Glpbio, USA). GAPDH was used as an internal reference for mRNA, and relative expression was quantified using the 2^-ΔΔCt^ method.

### Synthesis and characterization of hyaluronic acid hydrogels

Methacryloylated hyaluronic acid, sourced from Engineering for Life (China), was used to formulate a 5% hyaluronic acid gel. Initially, a 2.5% photofacial agent was resuspended in exosome precipitate (1.25 mg/ml concentration), and this suspension was combined with hyaluronic acid and stirred for 1 hour at room temperature. Following sterilization via a 0.22um sterile filter membrane, 200 ul of the mixture was transferred into a 1 ml syringe (tip removed). The mixture was then crosslinked with 395nm UV light for 6 seconds, yielding a cured exosome hydrogel. Finally, the gel was freeze-dried and its morphology was observed under a scanning electron microscope. To assess the biocompatibility of sodium hyaluronate hydrogel, an equal number of VK2 cells were seeded onto the hyaluronic acid hydrogel in a 96-well culture plate. After 24 hours of incubation, cell viability was measured using a live/dead staining kit (Beyotime, China) and the CCK-8 assay, following the manufacturer's instructions.

### Release of exosomes

A certain amount of exosomes was integrated into the hyaluronic acid hydrogel, which was then transferred to a 24-well plate containing 200 μL of PBS. The liquid was replaced every other day, and the supernatant was collected for BCA protein concentration measurements prior to each liquid change. The protein concentration determined represented the mass of exosomes released.

### Establishing a rat model of menopause

32 healthy 15-week-old female SD rats were purchased from Sipeifu Biotechnology (Beijing, China). These rats were randomly allocated into five groups (n = 6 in sham, ovariectomy, hydrogel, estrogen group; n = 8 in exosomes hydrogel group, 2 of them are marked with DIR). Oophorectomy surgery involved anesthetization using 1.25% tribromoethanol (250 mg/kg intraperitoneally), followed by an incision in the lower abdomen. In the model group, both ovaries were removed while in the sham operation group, some adipose tissues around the ovaries were excised. Antibiotics were administered post-operation to prevent infections.

Post-operation, vaginal smears were examined daily from the 10th day onwards for 5 days. The absence of a motility phase response indicated a successful menopause model. Hyaluronic Acid hydrogel loaded with exosomes was injected into the rats' vaginas, which were subsequently sutured to prevent gel loss. On the 3rd day, two rats implanted with DIR-labeled exosomes were sampled and processed for fluorescence microscopy. The remaining rats were euthanized on the 14th day, and vaginal tissues were collected for subsequent experiments.

### Hematoxylin-Eosin (HE) staining

Specimens were fixed with 4% paraformaldehyde, dehydrated with an alcohol gradient, cleared, and embedded in paraffin blocks. 5 μm thick tissue sections were then stained with hematoxylin/eosin (Absin, China) for routine histology.

### Immunohistochemistry (IHC)

Paraffin sections were deparaffinized and rehydrated through a series of xylene and alcohol washes. Endogenous peroxidases were removed with 3% hydrogen peroxide, and antigen retrieval was achieved with pH9.0 repair solution via autoclaving. Sections were then rinsed, dried, and incubated overnight at 4° C with primary antibodies (Ki67 and CD31, both 1:50 dilution, Absin, China). After rinsing, sections were incubated with a secondary antibody at 37° C for 20 min, and staining was developed with DAB. Quantification of staining in ten random fields per section was done with ImageJ software.

### Statistical analysis

Each experiment was performed three times and results are presented as mean ± standard deviation (SD). All statistical analyses were performed using GraphPad Prism 9. Comparisons between two groups were done with independent samples t-tests; comparisons among multiple groups were done using ANOVA. Differences were considered statistically significant at p < 0.05.
